# EMPATHY Life in Psoriasis: Embracing Patients’ Well-Being in Their Journey of Moderate-to-Severe Psoriasis

**DOI:** 10.3390/jcm13154469

**Published:** 2024-07-30

**Authors:** Francesca Prignano, Elena Campione, Aurora Parodi, Elena Vegni, Federico Bardazzi, Riccardo G. Borroni, Martina Burlando, Elisa Cinotti, Valentina Dini, Alfredo Giacchetti, Claudia Giofrè, Matteo Megna, Leonardo Zichichi, Maria Concetta Fargnoli

**Affiliations:** 1 Department of Health Sciences, Section of Dermatology, University of Florence, 50125 Florence, Italy; francesca.prignano@unifi.it; 2 Dermatologic Unit, Department of Systems Medicine, University of Rome Tor Vergata, 00133 Rome, Italy; campioneelena@hotmail.com; 3 Section of Dermatology, Dipartimento di Scienze della Salute, University of Genoa, Ospedale-Policlinico San Martino, IRCCS, Largo R. Benzi, 10, 16132 Genova, Italy; aurora.parodi@unige.it; 4 Department of Health Sciences, University of Milan, 20146 Milan, Italy; elena.vegni@unimi.it; 5 Unit of Clinical Psychology, ASST Santi Paolo and Carlo Hospitals, 20142 Milan, Italy; 6 Dermatology Unit, IRCCS Azienda Ospedaliero-Universitaria di Bologna, Policlinico S. Orsola Malpighi, 40126 Bologna, Italy; federico.bardazzi@aosp.bo.it; 7 Department of Biomedical Sciences, Humanitas University, 20089 Milan, Italy; 8 Dermatology Unit, Humanitas Research Hospital—IRCCS, 20089 Milan, Italy; 9 Dermatologic Clinic, DISSAL, San Martino Hospital, 16132 Genova, Italy; martinaburlando@hotmail.com; 10 Dermatology Unit, Department of Medical, Surgical and Neurological Sciences, University of Siena, 53100 Siena, Italy; elisacinotti@gmail.com; 11 Unit of Dermatology, University of Pisa, 56126 Pisa, Italy; valentinadini74@gmail.com; 12 UOC Dermatology, IRCCS INRCA, 60124 Ancona, Italy; alfredogiacchetti@gmail.com; 13 Dermatology Complex Operative Unit, Papardo Hospital, 98158 Messina, Italy; claudiagiofre@tiscali.it; 14 Section of Dermatology, Department of Clinical, Medicine and Surgery, University of Naples Federico II, 80138 Naples, Italy; mat24@libero.it; 15 Unit of Dermatology, San Antonio Abate Hospital, 80057 Trapani, Italy; dermatologia@asptrapani.it; 16 Department of Biotechnological and Applied Clinical Sciences, University of L’Aquila, 67100 L’Aquila, Italy; 17 Dermatology Unit, Ospedale San Salvatore, 67100 L’Aquila, Italy

**Keywords:** psoriasis, moderate-to-severe, empathy, patient well-being

## Abstract

**Background:** Psoriasis is a chronic inflammatory skin condition that affects millions of individuals worldwide, impacting their physical and emotional well-being. The management of psoriasis requires effective communication and a strong physician–patient relationship. **Objective:** We aim to develop a novel algorithm to enhance patient well-being and care in moderate-to-severe psoriasis, considering the time constraints that dermatologists have in public hospitals. **Methods:** This project employed a multidisciplinary approach, involving collaboration between 14 experienced dermatologists (referred to as Key Opinion Leaders: KOLs) and a psychologist. During three separate meetings (an initial virtual session, a face-to-face meeting, and a final virtual meeting), an algorithm (Embracing Patients’ Well-being in their Journey of Moderate-to-Severe psoriasis: EMPATHY), describing the patient’s reception through the entire first visit and follow-up visits, was developed and refined. **Results:** The EMPATHY algorithm provides a step-by-step approach from the moment the patient arrives at reception, through the first visit and on to subsequent visits. This algorithm fills a critical gap in the existing guidelines by specifically addressing how to foster empathy during psoriasis patient visits within time-limited consultations. The algorithm outlines patient-centered strategies at each visit. Key aspects include creating a welcoming environment, active listening, respecting privacy, tailoring communication styles, and managing patient expectations. **Conclusions:** The EMPATHY algorithm represents a novel and promising approach to improving patient care and well-being in moderate-to-severe psoriasis. Developed together by dermatologists and a psychologist, this algorithm offers healthcare providers practical guidance for managing both initial and follow-up patient visits. While further validation is necessary, the potential for adapting the EMPATHY algorithm to diverse healthcare settings and patient populations holds promise for improving patient outcomes across various chronic conditions.

## 1. Introduction

Psoriasis is an immune-mediated chronic inflammatory disease affecting approximately 2% of the population worldwide [1]. While it primarily manifests on the skin, causing erythema, scaling, pain, itching, and burning, psoriasis is now recognized as a systemic disease with significant extracutaneous complications [2,3]. Patients with psoriasis are at increased risk of comorbidities such as psoriatic arthritis, cardiometabolic syndrome, and inflammatory bowel disease [2]. Beyond physical symptoms, psoriasis can significantly impact a patient’s self-image and psychological well-being [4].

Visible lesions can lead to embarrassment, low self-esteem, anxiety, and depression, with some studies even reporting a link to suicide attempts [5,6,7,8,9]. The complexity of psoriasis necessitates a multidisciplinary and personalized approach to care [3,10]. Patients with psoriasis may benefit from a specialist coordination of care that addresses their overall mental, emotional, and physical health [11]. However, a notable gap still exists in terms of implementing this approach in routine clinical practice [12]. 

In recent years, significant progress has been made in understanding the complex mechanisms of psoriasis, leading to a diverse range of treatment options. Current management strategies focus on achieving remission of clinical manifestations, providing symptom relief, enhancing quality of life (QoL), and slowing disease progression [13,14]. These encompass various therapies such as topicals, phototherapy, systemic traditional medications, small molecules, and biologics targeting specific immune pathways [3]. However, challenges persist, including adverse effects, treatment resistance, safety concerns, and the financial burden [15]. 

For the successful long-term treatment of psoriasis, patient adherence is crucial. Several studies indicate a median adherence rate of only 50% among patients with chronic illnesses [16,17,18], potentially leading to misinterpretation of treatment failure. Moreover, treatment adherence significantly impacts on healthcare resources and patient outcomes [19,20]. Improving adherence rates could enhance health outcomes and reduce overall healthcare costs [21].

While treatment adherence is influenced by various factors [22], fostering patient–physician empathy can substantially enhance adherence rates and overall patient care. In the medical setting, empathy can be described as the cognitive ability to understand the patient, or as “feeling with” the patient [23,24]. Previous studies show that empathetic communication not only builds trust but also improves treatment adherence [18,21,25,26,27].

Our research group has actively investigated the role of empathy in improving patient care, particularly for chronic skin conditions like psoriasis. This focus stems from two key studies that we recently conducted. The first study, SHAring Patient Experiences (SHAPE) [28], which was a nationwide Italian survey, explored the challenges that patients face in achieving a good QoL with psoriasis. By gathering data from both dermatologists and patients, the SHAPE project revealed a gap between patients’ treatment desires and their perception of the disease. In particular, this study identified an important disparity with regard to the “expected” and the “reality” for aspects/items relating to well-being. Furthermore, approximately 40% of patients felt that their dermatologist was not considering their well-being and a similar proportion felt that their current treatment was inadequate for improving their signs and symptoms. When a lack of time can impact on the patient–physician relationship, having a simple but comprehensive questionnaire or algorithm can facilitate treatment choices and reduce patient delays. These findings suggest a potential underestimation of the social and psychological burden of psoriasis [28]. 

Building upon findings from SHAPE, a follow-up study [29] surveyed Italian dermatologists that were managing plaque psoriasis. This study found that while dermatologists prioritize QoL, patient satisfaction remains a challenge, indicating the need to better address patients’ holistic needs. The authors proposed the need for collaboration with psychologists and the development of tailored algorithms to enhance patient care by better aligning treatment goals with individual needs, ultimately improving compliance with treatment advice [29].

To date, there remains the notable lack of a comprehensive framework for managing the relationship between psoriasis patients and their physicians. While some protocols do exist, especially for inflammatory arthritic conditions, overall psoriasis management lacks sufficient guidance. Visalli et al. developed structured management pathways based on the close collaboration between hospital physicians, territorial specialists, and general practitioners. The overarching goal was to improve treatment adherence through a patient-centered approach and early diagnosis [30]. Another recent initiative, the Interdisciplinary Nurse-Coordinated SELf-MAnagement (INSELMA) intervention, targets inflammatory arthritis patients with a patient-centered approach [31], promoting shared decision-making and comprehensive care. Finally, a Belgian consensus defines a holistic treat-to-target (T2T) outcome [32], integrating physical and mental health parameters, including physician assessments and patient-reported outcomes.

The present algorithm, EMPATHY (Embracing Patients’ Well-being in their Journey of Moderate-to-Severe psoriasis), aims to address the need to establish an empathetic relationship between patients and dermatologists, ultimately enhancing the patient’s perception of the care provided and their relationship with the physician, thereby improving therapy adherence. Importantly, the adoption of this protocol is intended to enrich the patient–physician relationship while adhering to the standard timeframes for medical consultations.

## 2. Materials and Methods

### Project Design

**Figure 1:**
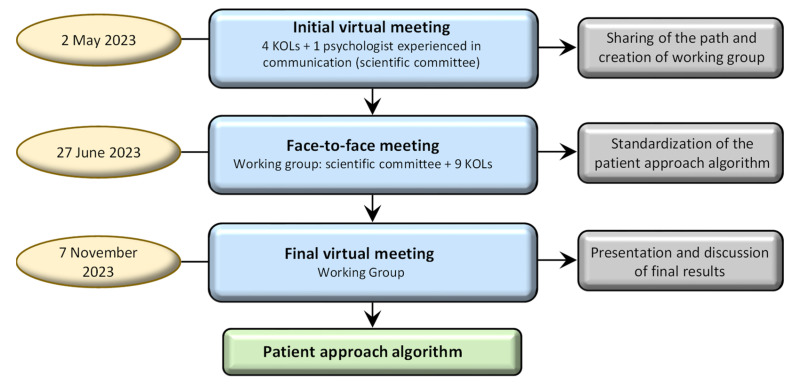
Project design. Abbreviations: KOLs = key opinion leaders.

This project was initiated with a virtual session on 2 May 2023. This was attended by 5 participants, comprising 4 Key Opinion Leader (KOL) dermatologists and 1 experienced communication psychologist, who together comprised the scientific committee (Figure 1). The purpose of this meeting was to establish a bi-disciplinary collaborative team framework, share the project’s journey, and create a working group. Following this, a face-to-face meeting took place on 27 June 2023 with 14 participants, including the scientific committee and the psychologist from the initial meeting, along with 9 other KOLs. During this session, the framework for patient engagement was meticulously outlined. A final virtual meeting was held on 7 November 2023, with the same attendees as the face-to-face meeting, to share and discuss the final results from the meeting.

## 3. Results

### 3.1. EMPATHY Algorithm

**Figure 2:**
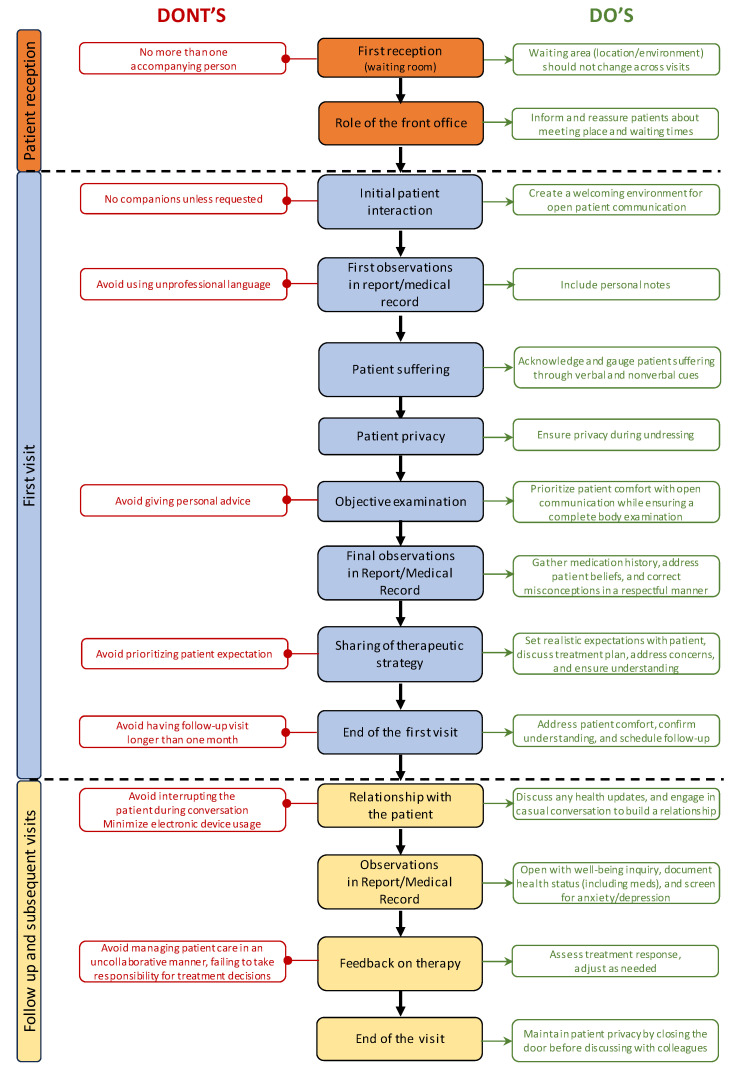
Simplified algorithm describing the patient’s reception through the entire first visit and follow-up visits. In the red boxes on the left, indicated by red lines with rounded ends, there are pieces of advice to pay attention to; on the right, in the green boxes indicated by green arrows, there are practical tips. In the center, in the colored boxes, are the main steps.

Following three separate meetings between KOLs and a psychologist, the EMPATHY algorithm was developed. It can be considered to be a novel approach to enhancing patient care and well-being in moderate-to-severe psoriasis. The entire algorithm is illustrated in Figure 2.

### 3.2. The First Visit

#### 3.2.1. Patient’s Reception

**Figure 3:**
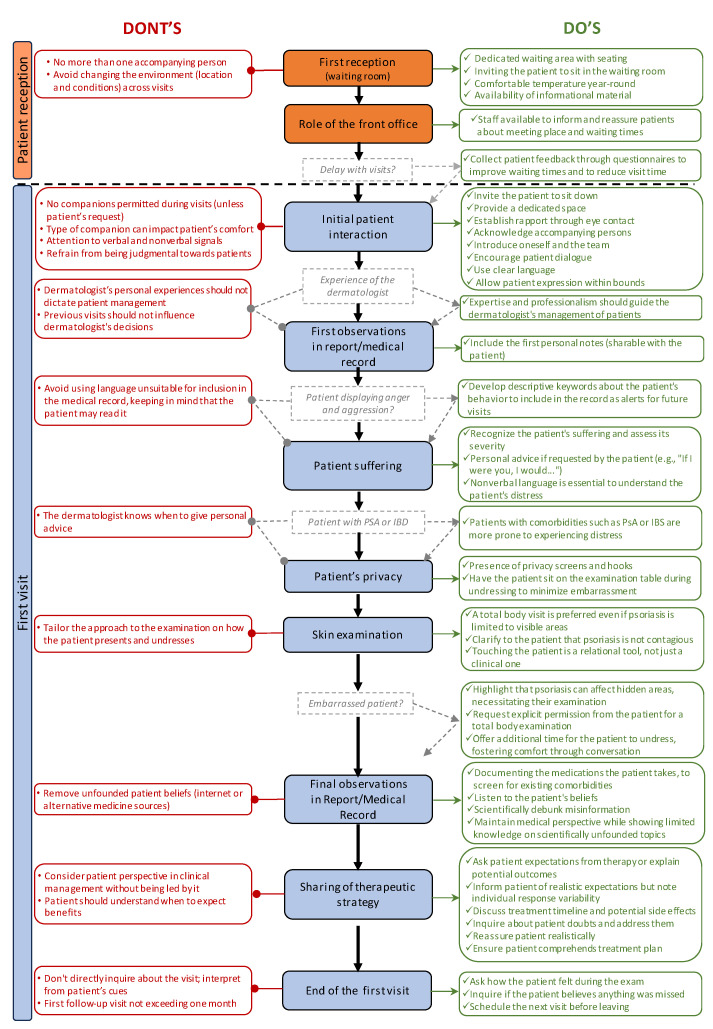
Additional information on the EMPATHY algorithm describing the patient’s reception through the entire first visit. In the red boxes on the left, indicated by red lines with rounded ends, there are pieces of advice to pay attention to; on the right, in the green boxes indicated by green arrows, there are practical tips. In the center, in the colored boxes, are the main steps. The gray boxes indicate a question that needs to be answered before proceeding. Abbreviations: PsA = psoriatic arthritis; IBD = inflammatory bowel disease.

The EMPATHY algorithm begins with the patient’s reception (Figure 3), which is a pivotal moment that can significantly impact upon the outcome of the final visit. The patient should be received in a comfortable waiting area equipped with seating and maintained at an appropriate temperature, that is, warm in winter and cool in summer. Scientific information should be made available to the patient, fostering a sense of familiarity and emphasizing their condition as the specialist’s focus.

A critical aspect is the front office. The staff (both nursing and non-nursing) responsible for the patient’s reception must be welcoming. They should provide information on waiting times and locations, and reassure patients (e.g., in the case of delays). Sometimes, especially in the case of appointment delays, it can be important to have patients fill out questionnaires, which can then be discussed during the actual visit (Figure 3). Implementing these questionnaires will serve to optimize dermatological visits, as physicians can simply comment on accurate responses without having to administer the questionnaire and wait for patients to complete them. Therefore, the initial approach, both in terms of the environment and staff greeting the patient, should be calm and welcoming, both physically in the waiting area and in terms of human interaction.

#### 3.2.2. Initial Patient Interaction: Creating a Supportive and Trustworthy Environment

In the initial patient interaction, clinicians play a crucial role in creating a supportive environment that fosters trust, communication, and collaboration. This begins with simple gestures, such as inviting the patient to sit down, and providing a dedicated space for consultations that ensures privacy and confidentiality. Establishing a bond through eye contact demonstrates respect for the patient’s individuality and support network. It is important to ignore the accompanying person in order to focus on the patient. Introducing oneself and the healthcare team humanizes the interaction, making the patient feel valued and understood. Patient dialogue should be encouraged through direct questions (e.g., “how are you?”). It is necessary to provide a general overview of the condition and ensure they have understood. Using clear, adapted language promotes comprehension and empowers patients in their healthcare journey. It is necessary to tailor the management of the visit and the manner of speaking according to the individual: researchers should observe the patient’s gestures and attire (how the patient presents themselves). Allowing patients to express themselves within appropriate boundaries validates their experiences and strengthens the therapeutic relationship. While companions can offer emotional support, clinicians should be mindful of their impact on the patient’s comfort and communication dynamics. It is necessary to allow the presence of only one accompanying person during the visit. Verbal and non-verbal cues, including facial expressions, can significantly influence the patient’s perception of the clinician and the overall quality of care.

Maintaining a non-judgmental attitude and avoiding bias are essential for building trust and fostering open communication. However, the dermatologist’s personal experiences also play a role in shaping their approach to patient care. Previous encounters with patients can leave an imprint, influencing clinical decisions in subsequent visits (for example, a negative experience with a drug can influence subsequent therapeutic choices for other patients). Thus, while expertise and professionalism remain crucial, an awareness of personal biases and the impact of past experiences is essential for providing optimal care.

In the initial observations reported within a report or medical record, it is essential to include personal notes that can be shared with the patient. When a patient exhibits anger or aggression, it is advisable to use descriptive keywords about their behavior for future reference without labeling them as “aggressive”. This approach ensures that the language used in the medical record is respectful and considerate of the patient’s perspective.

Recognizing the patient’s suffering and understanding its extent is crucial. When appropriate, engaging in “self-disclosure” can be a valuable tool. Some patients appreciate receiving personal advice from the dermatologist, as if the dermatologist were a family member (e.g., “If I were you, I would…”). However, it is crucial to exercise discretion and tailor self-disclosure to the individual patient’s preferences and cultural context. Additionally, gauging the patient’s distress through non-verbal cues plays a significant role in understanding their distress levels; typically, more visibly uncomfortable patients often bear a greater burden of suffering. It is to be noted that if we are dealing with a patient with comorbidities such as psoriatic arthritis or inflammatory bowel disease, it should be considered that they are more prone to experiencing distress that can extend beyond the skin.

Maintaining patient privacy during the initial visit is paramount. This can be offered, for example, by utilizing privacy screens, providing hooks for personal items, and allowing patients to sit on the examination table during undressing. Limiting mobile phone usage during consultations is advised to strengthen the therapeutic bond between the dermatologist and the patient, as well as with other team members and trainees.

#### 3.2.3. Skin Examination and Building Trust

Skin examination is another crucial moment for building a trusting relationship with the patient (i.e., building the patient–physician bond). Requesting a total-body examination, even if the psoriasis is limited to visible areas, demonstrates comprehensive patient care and fosters long-term patient loyalty. The importance of physical contact with the patient when undressed in enhancing trust and patient acceptance should be emphasized. However, it is essential to follow all infection prevention protocols, including using gloves during examinations, while also clarifying to the patient that psoriasis is not contagious. Touching the patient is a relational tool, not just a clinical one: it promotes confidentiality and mutual trust between the doctor and patient. It is vital to pay attention, however, because the approach to the examination should be tailored based on how the patient presents and undresses.

If the patient is embarrassed, explain that psoriasis can affect non-visible areas, including genital regions, which are important to examine as they can be predictive of psoriatic arthritis and other issues. This examination allows for the diagnosis of significant but often undisclosed localizations. It is important to explicitly ask the patient for permission for a total body physical examination.

Physicians should allow the patient a little more time to undress, and use chatting to put them more at ease. It is important to write down the medications that the patient is taking in the medical record to screen for present comorbidities and understand possible/contraindicated therapies; listen to the beliefs expressed by the patient and attempt to scientifically refute misinformation; maintain a medical standpoint by appearing less informed about scientifically unfounded topics; and pay attention to unfounded patient beliefs (i.e., information gathered from the internet or from naturopathy/homeopathy).

#### 3.2.4. Sharing Therapeutic Strategy and Conclusion of the First Visit

The therapeutic strategy is now shared with the patient. Therefore, it is important to ask the patient what they expect from the therapy or explain what can be expected from the treatment. It is useful to tell the patient what the concrete expectations may be, but it should be made clear that not all patients respond to the therapy in the same way. Discuss timelines (e.g., how long it is expected for the therapy to take effect, and how long it will be necessary to continue it). Discuss the potential adverse events of the therapy to prepare the patient and explain how to manage them if they occur. Ask the patient if they have any doubts and, if so, delve into them further. Reassure the patient realistically. Verify the patient’s understanding of the therapeutic plan: read the prescription together to ensure they have understood it well. Be guided by the patient’s psychological profile in choosing the biological treatment: the psychological profile matters! Pay attention to considering the patient’s point of view in their clinical management but do not be guided by it. The patient must understand when they can expect benefits by.

At this point, the visit ends. Ask the patient how they felt during the objective examination and if they think anything was overlooked during the objective examination. Thereafter, schedule the next visit at the end of the first visit, but be careful. Do not directly ask “how was the visit?”; deduce it from the patient’s verbal and non-verbal communication. Finally, the first follow-up should be very close to the first visit (e.g., after 1 month) to consolidate the emotional relationship between the dermatologist and patient and to create continuity in the patient’s journey. Subsequent follow-ups can be spaced further apart.

### 3.3. Follow-Up and Subsequent Visits

#### 3.3.1. The Patient Relationship

**Figure 4:**
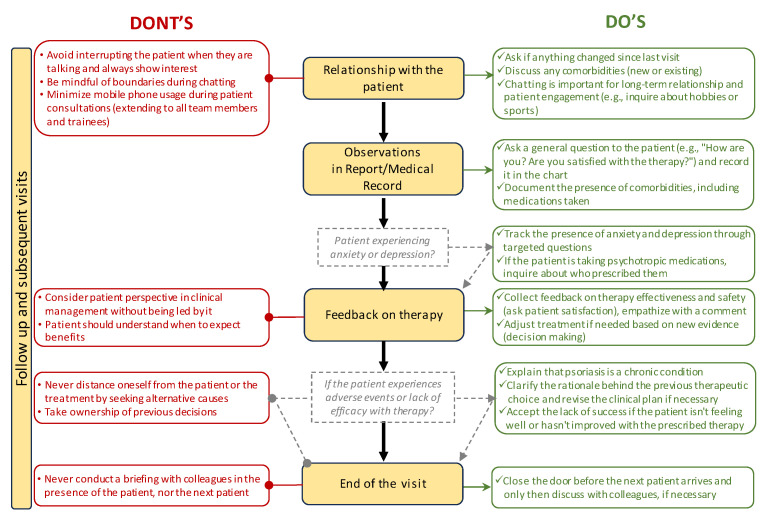
Additional information on the EMPATHY algorithm describing the follow-up and subsequent visits. In the red boxes on the left, indicated by red lines with rounded ends, are pieces of advice to pay attention to; on the right, in the green boxes indicated by green arrows, are practical tips. In the center, in the colored boxes, are the main steps. The gray boxes indicate a question to answer before proceeding.

In subsequent visits, it is important to nurture the patient relationship (Figure 4).

To enhance the therapeutic alliance, inquire if anything has changed since the last visit. Once a trust bond has been established with the patient, explore any comorbidities (whether new or pre-existing). Engage in casual conversation to build a long-term relationship and connect with the patient (e.g., inquire about hobbies or sports activities). Discussing comorbidities reassures the patient and demonstrates genuine interest in their well-being. Active listening is crucial for building rapport and understanding the patient’s perspective. While it is essential to allow patients to express themselves freely, effective communication also involves guiding the conversation to ensure a focused and productive dialogue. This may involve gently redirecting the patient when necessary to stay on track and address their primary concerns. Be mindful of boundaries during casual conversations!

#### 3.3.2. Observations in Medical Records

Pose a general question to the patient (e.g., “how is their mood?”; “how are they feeling in general?”; “how satisfied are they with the therapy?”) and document it in the medical records. If the patient experiences anxiety or depression, track their presence through targeted questions. In cases where the patient takes psychotropic medications, inquire about their prescriber.

#### 3.3.3. Feedback on Therapy

Gather feedback on the effectiveness and safety of the ongoing therapy (e.g., ask the patient how it is going, and if they are satisfied with the therapy), and provide a commentary in order to empathize with the patient. If the patient is not feeling well or has not improved with the prescribed therapy, make appropriate decisions based on new evidence (i.e., decision-making). If the patient has experienced adverse events or a lack of efficacy with the prescribed therapy, explain that psoriasis is a chronic disease, clarify the rationale behind the previous therapeutic choice, and reformulate the clinical plan if necessary. Accept failure if the patient is not feeling well or has not improved with the prescribed therapy (avoid referring to it as a failure but rather as an opportunity to start anew). Be careful never to distance oneself from the patient or the treatment by seeking alternative causes—take ownership of previous decisions.

### 3.4. End of Visit

Close the door before the next patient arrives and only discuss information with colleagues at this point, if necessary. Never brief colleagues in the presence of the patient or the next patient.

## 4. Discussion

The EMPATHY algorithm represents an innovative approach that is aimed at enhancing the overall well-being perception of patients with moderate-to-severe psoriasis through empathetic communication with physicians throughout the entirety of the patient’s journey. Developed through collaboration between dermatologists and a psychologist, it serves as a valuable tool for managing patients’ initial visits and follow-up appointments within the typical time constraints of public hospitals.

A review of the literature revealed that effective communication and shared decision-making between patients and healthcare providers are crucial for the management of psoriasis [33,34,35,36,37]. There is a recognized need for improvement in dermatologist–patient communication, particularly regarding treatment discussions and addressing patients’ emotions and concerns [33,34]. Barriers to shared decision-making include patients’ misconceptions, time constraints during consultations, and the need for continuity of care with a regular physician [35]. Despite challenges, patients value individualized care and trust in their physicians’ expertise but desire that more personalized attention be paid to their emotional well-being [33,36]. In addition, dermatologists’ personal beliefs and assumptions about patients may influence their prescribing behavior and shared decision-making practices, indicating a need for additional training and education to enhance patient-centered care [36].

Building upon these insights from the literature, the EMPATHY algorithm emphasizes a patient-centered approach to psoriasis care, whereby doctors listen to patients’ concerns, build empathy with the patient, and collaborate with them to develop a treatment plan. Recent studies have reported protocols or useful indicators to enhance psoriasis patient management. Helliwell et al. reported eight best-practice indicators across four key focus areas aligned with the patient pathway; however, among these, no guides were reported to improve the relationship between physicians and patients [38]. Visalli et al. [30] propose a patient-centered approach aimed at improving access to care, ensuring appropriate treatment, and effectively monitoring progress. In this project, the presence of a psychologist in the multidisciplinary team is advocated. In the INSELMA approach, Health Care Professionals (HCPs) work together with patients to understand their beliefs and what matters most to them [31]. HCPs show that they care by being actively involved, listening with empathy, and making decisions together about treatment. They aim to provide complete care that addresses all aspects of the patient’s situation. However, the way HCPs behave can sometimes hamper a patient’s ability to manage their own health. This may occur if they blame the patient, make them feel bad, or set unrealistic expectations. The Belgian T2T Algorithm for psoriasis management requires collaborative decision-making between physicians and patients to emphasize treatment adherence [32]. It identifies four primary domains, each with predefined subitems outlining ideal and acceptable targets. Among these, the well-being domain is focused on measuring the impact on daily activities through patient-reported outcomes.

A crucial factor in influencing treatment adherence in psoriasis patients is the empathetic relationship with the physician [22]. A study by Fischer et al. found a clear link between patient dissatisfaction and poor adherence [39]. Similarly, Van de Kerkhof et al. demonstrated the importance of the patient–physician relationship in managing psoriasis [40]. In a recent systematic review conducted by Larsen et al., patients emphasized the importance of being treated as individuals [35], highlighting that the impact of living with psoriasis extends beyond the skin [41]. They expressed frustration at the belief that those without psoriasis, including doctors, cannot fully comprehend the disease’s effects [36]. Patients highly appreciated practitioners who demonstrated knowledge, empathy, and a willingness to discuss psoriasis care [36,42]. However, the literature suggests that this ideal scenario is often not realized. Patients reported losing faith in their doctors when they felt that their concerns were not taken seriously, negatively impacting adherence and the patient–provider relationship [36,42]. A systematic review by Larsen et al. [35], exploring decision-making in psoriasis research, reveals a significant emphasis on communication. Patients consistently express a strong desire to be actively heard and to establish meaningful rapport with their healthcare providers. Patients also desired physicians to use non-technical language, pay closer attention to their input during consultations, and demonstrate greater sensitivity to their experiences [35].

In this scenario, the EMPATHY algorithm addresses the unmet need for recommendations on managing psoriasis patient visits, providing specific instructions on how to foster empathy with patients. It outlines the attitudes that healthcare providers should adopt to connect with patients and those to avoid, ensuring that patients feel heard and valued. The algorithm emphasizes the importance of communication and patient-centered care, optimizing the visit within the standard 15–20 min timeframe. Future plans include validating the algorithm for potential application in managing other chronic conditions.

## 5. Study Limitations

The EMPATHY algorithm is a hypothesis-generating study. It has not been validated or tested. We are currently planning to validate this algorithm in a separate study involving dermatologists and patients with psoriasis (with diverse backgrounds) to evaluate its effectiveness in improving patient outcomes. Adapting the algorithm to different healthcare settings and patient populations might necessitate adjustments, potentially introducing challenges in maintaining standardization.

## 6. Conclusions

The EMPATHY algorithm emphasizes empathy and effective communication between the physician and patient. It aims to improve the QoL of psoriasis patients by focusing on both the disease itself and their psychological and social well-being. The algorithm can be adapted to suit different healthcare settings and patient populations. Ongoing research and validation can further enhance its effectiveness.

## Data Availability

No data were generated in the present project.
